# Public Health: Back to the Future

**DOI:** 10.3389/fpubh.2013.00012

**Published:** 2013-04-30

**Authors:** Richard Reid Clayton

**Affiliations:** ^1^Department of Health Behavior, College of Public Health, University of KentuckyLexington, KY, USA

This book is dedicated to and honors the signal career accomplishments and the large professional and personal impact F. Douglas Scutchfield, MD, MPH has had on medicine and public health. Holsinger describes Scutch as a “doctor's doctor and a public health practitioner's practitioner.” In many ways Scutch has become an icon in medicine and public health, a larger than life innovator and tour-de-force much like his early mentor and hero, William R. Willard, MD.

This book contains 11 crisply written, comprehensive, and very interesting substantive chapters. The first chapter by Steven Woolf and Paula Braveman provides a cogent overview of the role of social determinants on health disparities. Anyone reading this chapter will come away with a much better understanding of how macro-level factors frame the issues that must be dealt with to constructively address a health/sick care system that is unacceptably costly, inefficient, and often irrational.

Ingram, Costich, and Perez describe in elegant detail the factors influencing poor health in marginalized populations and geographic places that have shaped the life and work of Doug Scutchfield (i.e., Appalachia where he born and raised, the Black Belt of Alabama, and Barrio Logan in San Diego). These authors conclude with a clinical and socioecological perspective and a description of a solution to the health of marginalized populations crafted by the Robert Wood Johnson Foundation under the leadership of Debra Perez. For significant reductions in indices of morbidity and mortality, the health of various population groups require an educated public health workforce. Evashwick in Chapter 3 discusses the recent growth in the number of schools of public health and other educational programing trends. The bulk of the chapter deals with workforce issues (i.e., shortages, alignment of skills and needs, inter-professional collaboration, global health, cultural competence and health literacy, and teaching technologies and innovations). Reflecting the heterogeneity that exists in public health in the twenty-first century, she says: “Currently, the public health workforce in the United States is like a brightly colored Rubik's cube that can be organized and viewed from different perspectives for different purposes.” While this metaphor captures well numerous ways to view the public health workforce, the reference to Rubik's cube is unfortunately dated. In an era when cost, comparative effectiveness, and emphasis on prevention demand attention, Matheny deftly addresses the issues of community-oriented primary care from both a medical and public health perspective. He ends with a re-affirmation of the critical role that patient-centered medical homes will play under the Affordable Health Care Act. There will be increased pressure on family care practitioners to collaborate and coordinate patient care with multidisciplinary team members from health professions. A balanced mixture of clinical medicine and public health practice will be required for improving outcomes and the process of delivering quality care. Addressing the issue of who is the public in public health, Mathews does a good job of describing the relevance of democratic process (naming, framing, deliberative decision-making, identifying and committing resources, organizing complementary, and public learning) that must be present in Affordable Care Communities. Mays, Halverson, and Riley in Chapter 6 provide an excellent review of the history, development, current status, and future opportunities and challenges of public health services and systems research (PHSSR), an area of public health and medicine in which Doug Scutchfield's footprint is deep and large. A synoptic review of a taxonomy of seven local health system configurations published by Mays, Scutchfield, and their colleagues in 2010 and an example to help understanding of variation in public health spending is a highlight of this chapter. In an excellent chapter, Bender describes the development, process, standards and measures, and the twelve domains of accreditation for public health departments. Accreditation of public health departments is an innovation whose time has arrived and is a critical part of professionalization that will enhance the evidence-based practice of public health throughout the country. Seidman, Silberg, and Patrick's chapter on contemporary issues in scientific communication and public health education is a comprehensive review of an area that spans a number of academic and practice disciplines. It devotes considerable attention to the importance of new technologies and their potential impact on public policy. The chapter by Wyatt, Brady, and Maynard is a succinct summary of best practices in establishing and maximizing the power of partnerships in public health. In Chapter 10, Halverson, Mays, and Hogg describe in detail the “systemness” in public health with special attention to the components of public health at the state and federal levels, and the challenges faced by these systems in the second decade of the twenty-first century. The chapter by Schoenbaum, Osborn, and Squires uses data from a global perspective to identify lessons for the United States on health, health care, and health policy. The unique, Federal form of government and delegation of responsibility for health and health care functions at the state level has contributed to the lack of coherent national policies. These authors see the Affordable Care Act as possibly a first step toward “…more national, or at least more nationally coordinated, approaches to health and health care policy.”

The inner core of this book consists of clearly written, seamlessly connected, informative, and critical yet positive chapters that describe public health systems and services that are encouraging. They are bracketed by an introduction by Holsinger and Scutchfield and a concluding chapter on the future of public health by Keck, Scutchfield, and Holsinger that together provide perhaps the best overview of public health and health care in existence. This is a book that anyone and everyone in public health should read (from students in undergraduate, graduate, professional education to those providing health to the public in various sized local health departments, to members of boards of health to public policy makers). It captures extremely well the many strengths, opportunities, and challenges that are at the core of public health functions and services in public health systems. In addition, this book describes in a persuasive way why those involved in creating evidence-based practice and practice-based evidence are so committed to the insuring the “good of the order.”

## Conflict of Interest Statement

Drs. Holsinger, Scutchfield, Mays, Costich, Ingram and Wyatt are my colleagues in the University of Kentucky, College of Public Health. My review of this book has not been influenced in any way because I know them. Therefore, I don't believe any potential conflict of interest exists.

